# Systemic Inflammation Index (SII) as a Predictor of Mortality in Intensive Care Units

**DOI:** 10.3390/biomedicines13071669

**Published:** 2025-07-08

**Authors:** Ömer Emgin, Elif Rana Kılıç, İmren Taşkıran, Engin Haftacı, Adnan Ata, Mehmet Yılmaz

**Affiliations:** Intensive Care Unit, Kocaeli City Hospital, Kocaeli 41060, Turkey

**Keywords:** intensive care, mortality, neutrophil-to-lymphocyte ratio (NLR), platelet-to-lymphocyte ratio (PLR), Systemic Inflammation Index (SII)

## Abstract

**Background:** The Systemic Inflammation Index (SII), associated with increased systemic inflammation and adverse outcomes, has been demonstrated to be efficacious and a significant biomarker in different patient populations. This investigation aims to examine the correlation between the admission SII, a relatively new biomarker, and 28-day mortality outcomes in intensive care units (ICUs). **Methods:** This retrospective cohort analysis was undertaken in a tertiary-level ICU in Turkey from 3 April 2024 through 31 December 2024. Baseline demographic data, clinical characteristics, and laboratory parameters were recorded. Inflammatory parameters such as SII, NLR, and PLR were calculated at the time of ICU admission. SII = neutrophil count (10^3^/ill) × PLT count (10^3^/μL)/lymphocyte count, NLR = neutrophil count (10^3^/μL)/lymphocyte count (10^3^/μL), and PLR = PLT (10^3^/μL)/lymphocyte count (10^3^/μL). **Results:** In this study, a total of 702 patients who met the eligibility criteria were recruited. The study’s overall mortality rate for 28 days was 36.9% with 259 deaths. The median age of the cohort was 70 years (57–80), with 41.6% of the participants being female. The SII was markedly elevated in non-survivors compared to survivors (*p* = 0.010). The analysis revealed that the SII/1000 was an independent predictor of elevated mortality risk (OR 1.029, 95% CI 1.001–1.057, *p* = 0.042). **Conclusions:** The identification of the Systemic Inflammation Index on admission to the ICUs is of critical importance. The SII has been demonstrated to serve as a significant and independent predictor of mortality. There is a need for prospective and large-scale studies to generalize this finding to other populations or for more widespread use in clinical practice.

## 1. Introduction

Critically ill patients in intensive care units (ICUs) have a high mortality rate [1]. Clinicians need appropriate prognostic biomarkers to optimize clinical management and resource allocation in predicting mortality in ICUs [2,3]. Recent studies have indicated that the Systemic Inflammation Index (SII), a relatively new biomarker, may be a valuable predictor of outcomes for critically ill patients [4]. The SII is derived from routine hematological parameters (platelet, neutrophil, and lymphocyte counts) [2]. The ability to derive the SII from routine complete blood count (CBC) tests may improve its practicality and suitability for widespread clinical use [2,4,5]. It provides a composite measure of immune and inflammatory activity [6]. The SII, associated with increased systemic inflammation and adverse outcomes, has been demonstrated to be efficacious and a significant biomarker in different patient populations (oncology, neurology, cardiovascular disease, and sepsis) [7,8,9,10,11,12]. There are limited studies examining the relationship between SII and mortality from all causes in critically ill patients.

Inflammation is a vital component of the body’s natural defense mechanisms. Dysregulation of this critical mechanism or excessive inflammatory activation can lead to immune paralysis, tissue injury, and organ dysfunction. After any situation that triggers the immune system, patients often experience a systemic inflammatory response syndrome (SIRS), followed by a compensatory anti-inflammatory response syndrome (CARS) [13]. Therefore, maintaining a balance between immune system activation and suppression is crucial for the management of critically ill patients. Conventional biomarkers such as the neutrophil-to-lymphocyte ratio (NLR) and the platelet-to-lymphocyte ratio (PLR), C-reactive protein (CRP), and procalcitonin have also been utilized as prognostic markers in ICU patients. However, their limitations, such as insufficient specificity and failure to adequately represent the dynamic balance between immune activation and suppression, highlight the need for more comprehensive indicators [2,14].

Systemic inflammation in ICU patients is often exacerbated by preexisting comorbidities, invasive procedures, and prolonged hospitalization. In this context, the SII is a practical and insightful tool to assess the inflammatory burden and its potential impact on patient prognosis [9]. In addition, its predictive utility may support timely and informed clinical decisions, such as the implementation of targeted anti-inflammatory strategies or enhanced monitoring of patients at increased risk [6].

This study aims to assess the association between the admission SII, a relatively novel biomarker, and 28-day mortality in ICU patients.

## 2. Materials and Methods

**Figure 1:**
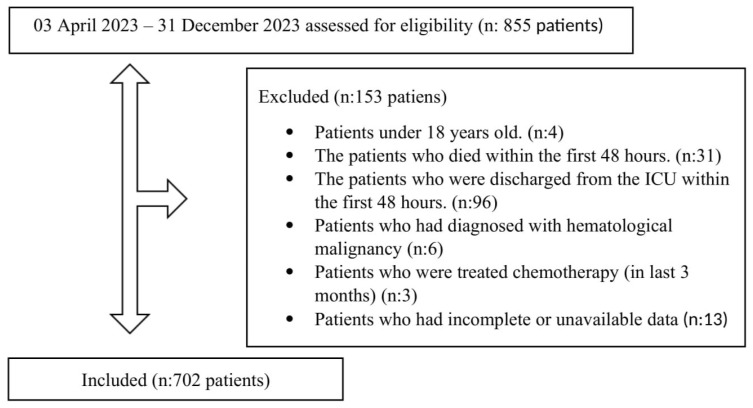
Flowchart of Study.

This retrospective cohort analysis was undertaken in a tertiary-level ICU in Turkey from 3 April 2024 through 31 December 2024. The study protocol received approval from the Kocaeli City Hospital Local Ethics Committee (Approval No: 2024/7, Date: 15 January 2024). A total of 805 patient records were reviewed for the study. Inclusion criteria required patients to be adults (≥18 years) admitted to the ICU for at least 48 h, with complete hematological and clinical data available at admission. Exclusion criteria were carefully defined to ensure a homogeneous cohort and minimize confounding factors: patients staying in the ICU for less than 48 h were excluded to focus on critically ill patients requiring prolonged ICU management, as short stays may reflect less severe conditions or rapid transfers [15,16,17]. Patients with hematological malignancies were excluded due to their altered hematological profiles, which could skew SII calculations, as these conditions directly affect neutrophil, lymphocyte, and platelet counts [9,14]. Patients treated with chemotherapy within the last 3 months were excluded because chemotherapy can induce immunosuppression or bone marrow suppression, significantly altering SII parameters and confounding its prognostic value [14]. In contrast, patients with non-hematological malignancies were included, as their inflammatory profiles are less likely to directly interfere with SII calculations, and their inclusion allows for a broader representation of critically ill patients with comorbidities commonly encountered in ICUs [9]. After exclusions, 702 patients were included in the final analytical cohort (Figure 1).

Baseline demographic data, clinical characteristics, and laboratory parameters collected at the time of ICU admission were recorded. Patient characteristics, such as age, gender, underlying diseases, organ failure score, mortality prediction score, Charlson Comorbidity Index (CCI), the main cause of ICU admission, Acute Kidney Injury (AKI), hemodialysis treatment, length of stay in ICU (LOS-ICU), mechanical ventilation support, and 28 and 90-day mortality were recorded.

Laboratory parameters analyzed on admission (within 24 h) included complete blood counts, coagulation profiles, serum electrolytes, liver and kidney function tests, inflammatory markers (CRP and procalcitonin), and arterial blood gas parameters. Inflammatory parameters such as SII, NLR, and PLR were calculated. SII = neutrophil count (10^3^/μL) × PLT count (10^3^/μL)/lymphocyte count, NLR = neutrophil count (10^3^/μL)/lymphocyte count (10^3^/μL), and PLR = PLT (10^3^/μL)/lymphocyte count (10^3^/μL).

Data are presented as percentages, mean ± standard deviation, or median with interquartile range. Categorical variables were analyzed using the chi-square test, while normally distributed continuous variables were compared using Student’s *t*-test. Non-parametric continuous variables were compared between groups using the Mann–Whitney U test. Statistical significance was considered for *p*-values ≤ 0.05. Multivariate logistic regression was performed, incorporating the SII and other covariates that reached statistical significance (*p* < 0.05) in univariate testing. An adjusted odds ratio (OR) and a 95% confidence interval (CI) were reported for each independent factor. Model fit was assessed using the Hosmer–Lemeshow goodness-of-fit test, and explanatory power was evaluated with Nagelkerke R^2^. All statistical analyses were performed using IBM SPSS Statistics version 26.0 (IBM Corp., Armonk, NY, USA).

## 3. Results

A total of 855 patients were assessed for eligibility between 3 April 2023 and 31 December 2023. Among these, 153 patients were excluded for the following reasons: age under 18 years (n:4), death within the first 48 h (n:31), discharge from the ICU within the first 48 h (n:96), diagnosis of hematological malignancies (n:6), chemotherapy treatment within the last 3 months (n:3), and incomplete or unavailable data (n:13). After exclusions, 702 patients were included in the final analytical cohort (Figure 1).

The study’s overall mortality rate for 28 days was 36.9% with 259 deaths. The median age of the cohort was 70 years (57–80), with 41.6% of the participants being female. Non-survivors had a significantly higher median age compared to survivors (*p* < 0.001). The most comorbidities were hypertension (HT) n:338 (48.1%), diabetes mellitus (DM) n:219 (31.2%), congestive heart disease (CHD) n:200 (28.5%), malignancy n:165 (23.5%), and coronary artery disease (CAD) n:136 (19.4). The non-surviving patients had a significantly higher ratio of HT, CHD, malignancy, CAD, pulmonary disease, and kidney disease compared to the surviving patients (*p* < 0.025, <0.001, 0.003, <0.001, <0.001, and <0.001, respectively). The main reason for ICU admission was postoperative patients (35.2%), followed by sepsis (25.9%), neurological causes (11.7%), respiratory causes (11.5%), trauma (9.0%), and other causes (5.6%). Sepsis was the most common admission cause of ICU among non-survivors and was statistically significantly higher than survivors (40.5%, *p* < 0.001). Postoperative conditions were more common in survivors (43.3%, *p* < 0.001). The average APACHE-II score for all patients was 19.97 ± 8.30. The non-survivor group exhibited significantly higher APACHE-II scores, SOFA scores, and CCI (*p* < 0.001 for all). The non-survivor group had significantly higher ratios of AKI, hemodialysis treatment, and invasive mechanical ventilation (IMV) treatment (*p* < 0.001 for all). The survivor group had significantly higher ratios in duration of IMV day, and length of hospital stay (*p* < 0.001 for all). The overall mortality for 90 days was n:332 (47.3) (Table 1).

Admission laboratory parameters were compared between the survivor and non-survivor groups. Non-survivors demonstrated significantly elevated leukocyte and neutrophil counts compared to survivors (*p* < 0.001 for both). Hemoglobin levels, lymphocyte counts, and platelet counts were significantly lower in non-survivors than in survivors (*p* < 0.001, 0.009, 0.001, respectively). Creatinine, albumin-corrected calcium, magnesium, and phosphorus levels were all significantly higher in non-survivors than in survivors (*p* < 0.001, 0.001, 0.001, <0.001, respectively). Aspartate aminotransferase (AST) and alanine aminotransferase (ALT) levels were significantly higher in non-survivors than in survivors (*p* < 0.001 and *p* < 0.034, respectively). Non-survivors had significantly lower levels of total protein (5.46 g/dL) and albumin (2.80 g/dL) than survivors (5.77 g/dL and 3.20 g/dL, respectively; *p* = 0.001 and *p* < 0.001, respectively). pH levels were lower in non-survivors (7.36) compared to survivors (7.40) (*p* < 0.001), and lactate levels were significantly higher in non-survivors (2.03 mmol/L vs. 1.55 mmol/L in survivors; *p* < 0.001) (Table 2).

Comparative analysis of inflammatory markers between survivors and non-survivors is presented in Table 3. The SII was significantly elevated in non-survivors compared to survivors (*p* = 0.010). Similarly, non-survivors had significantly higher NLR values (*p* < 0.001). In contrast to SII and NLR, the PLR failed to discriminate between survivors and non-survivors (*p* = 0.326) (Table 3).

The multivariate logistic regression analysis identified significant risk factors for mortality. Higher APACHE-II scores (OR 1.065, 95% CI 1.028–1.102, *p* < 0.001), SOFA scores (OR 1.202, 95% CI 1.107–1.304, *p* < 0.001), CCI (OR 1.111, 95% CI 1.064–1.160, *p* < 0.001), lactate levels (OR 1.156, 95% CI 1.053–1.269, *p =* 0.002), and SII (OR 1.029, 95% CI 1.001–1.057, *p =* 0.042) were significantly associated with increased mortality risk. CRP (OR 1.002, 95% CI 1.000–1.004, *p =* 0.051) and creatinine (OR 1.097, 95% CI 0.937–1.283, *p =* 0.251) were not statistically significant. The model showed a good fit to the data (Hosmer–Lemeshow test: χ^2^ = 10.393, df = 8, *p =* 0.239) and moderate explanatory power, explaining approximately 36.6% of the variance in mortality (Nagelkerke R^2^ = 0.366) (Table 4).

## 4. Discussion

It is essential to identify effective and accessible biomarkers in patients in ICUs in order to effectively manage their illnesses and optimize resource allocation. Inflammation is a common and significant phenomenon in this group, with a high mortality rate [9]. This retrospective cohort study aimed to evaluate the association between the SII at ICU admission and 28-day all-cause mortality among critically ill patients. The results of this study demonstrated that the SII at the time of admission can serve as an effective indicator of all case mortality in ICUs. The SII was further identified as an independent risk factor for 28-day mortality.

Inflammation is common and essential for critically ill patients; however, it must be balanced. If the body’s response to inflammation is dysregulated or excessive, it will be harmful to the body and result in organ failure [18]. This situation has the potential to further increase the risk of death for intensive care patients, who already exhibit a high mortality rate [19]. Intensive care specialists need biomarkers to predict mortality rate for optimizing resources of ICUs and balanced treatment of inflammation in critically ill patients. Previous studies demonstrated that inflammatory biomarkers (procalcitonin, CRP, NLR, PLR) were associated with mortality in the ICUs. However, they had some limitations [2,20]. Recent studies have indicated that the SII may be a more comprehensive indicator of mortality in critical patients suffering from sepsis and confirmed cases of COVID-19 [4,5,8]. The SII, which incorporates neutrophils, lymphocytes, and platelets against PLR and NLR, has the potential to assist clinicians in more accurately diagnosing and managing critically ill patients. The fact that it is easily accessible, inexpensive, and can be calculated from routine blood counts also increases the importance of the SII. It may be hypothesized that repeated measurements of SII during intensive care will contribute to disease management, and studies can be designed to test this hypothesis.

Zhan et al.’s study (2023) demonstrated that SII is a significant parameter in determining prognosis in patients with aortic aneurysm [21]. In addition to APACHE 2 and SOFA scores, which are frequently used to predict mortality and morbidity in intensive care patients, the independent statistical significance of the CCI, which we included in this study, demonstrated the usability of this parameter in all patients. As demonstrated in the extant literature, elderly patients and those with comorbidities exhibit an elevated mortality rate in intensive care settings. In the present study, data analysis revealed that the non-survivor group was characterized by higher ages and the presence of comorbidities [4,18,22]. The effective management of comorbidities, in parallel with the primary reasons for admission to intensive care, will reduce mortality rates. This emphasizes the significance of multidisciplinary follow-up in intensive care [23].

Sepsis is a prevalent syndrome that is associated with a high mortality rate in ICUs [24]. In this study, it was identified as the primary cause of admission for patients who did not survive. In septic patients, the characteristic hematological profile includes neutrophilia and thrombocytosis accompanied by lymphopenia. Consequently, SII, which integrates these parameters, may be considered a potential predictor of sepsis-related mortality. It was observed that liver and kidney dysfunctions were also manifested in non-survivors. However, due to the retrospective design of this study, significant differences in other parameters between the survivor and non-survivor groups limit the generalizability of the findings. There is a critical need for prospective, standardized studies, particularly in sepsis patients, to validate and extend these observations. The study demonstrates the necessity of developing treatment strategies and increasing training for sepsis. The IMV treatment has been associated with high mortality in intensive care patients [3,25]. However, this treatment was found to be less common in non-survivors in this study. Furthermore, the LOS-ICU was found to be significantly shorter in non-survivors. These results may be attributable to the fact that patients with more severe disease scores were lost at an early phase.

This study had some limitations. It was a retrospective and single-center study. Additionally, we recorded the SII only at admission. In the event of protracted hospitalization, it may become necessary to re-evaluate the levels of inflammatory parameters. Nevertheless, the cohort’s primary strengths lie in its substantial sample size and its comprehensive assessment of all-cause mortality.

## 5. Conclusions

The identification of the Systemic Inflammation Index on admission to the ICUs is of critical importance. The SII has been demonstrated to serve as a significant and independent predictor of mortality. There is a need for prospective and large-scale studies to generalize this finding to other populations or for more widespread use in clinical practice. Additionally, it may be important to evaluate this parameter with repeated measurements, especially during prolonged intensive care stays.

## Figures and Tables

**Table 1:** Baseline demographic data and clinical characteristics comparison between survivors and non-survivors.

	All Patients n:702 (100%)	Survivors n:443 (63.1%)	Non-Survivors n:259 (36.9%)	*p*-Value
Age (median years)	70 (57–80)	69 (55–79)	73 (61–81)	**0.001**
Gender (n (%))				
Female	292 (41.6)	194 (43.8)	98 (37.8)	0.112
Male	410 (58.4)	249 (56.2)	161 (62.2)	
Comorbidities (n (%))				
Hypertension	338 (48.1)	199 (44.9)	139 (53.7)	**0.025**
Diabetes Mellitus	219 (31.2)	127 (28.7)	92 (35.5)	0.059
Congestive Heart Disease	200 (28.5)	106 (23.9)	94 (36.3)	**<0.001**
Malignancy	165 (23.5)	88 (19.9)	77 (29.7)	**0.003**
Coronary Artery Disease	136 (19.4)	66 (14.9)	70 (27)	**<0.001**
COPD	112 (16.0)	54 (12.2)	58 (22.4)	**<0.001**
Cerebrovascular Disease	108 (15.4)	69 (15.6)	39 (15.1)	0.854
Chronic Kidney Disease	73 (10.4)	32 (7.2)	41 (15.8)	**<0.001**
Liver Disease	6 (0.9)	2 (0.5)	4 (1.5)	0.129
Causes of ICU Admission (n (%))				
Sepsis	182 (25.9)	77 (17.4)	105 (40.5)	**<0.001**
Respiratory causes	81 (11.5)	47 (10.6)	34 (13.1)	0.314
Neurological causes	82 (11.7)	53 (12.0)	29 (11.2)	0.760
Trauma	63 (9.0)	46 (10.4)	17 (6.6)	0.088
Postoperative patients	247 (35.2)	192(43.3)	55 (21.2)	**<0.001**
The other causes	39 (5.6)	23 (5.2)	16 (6.2)	0.582
First day diagnosis/treatment (n (%))				
Acute Kidney Injury	100 (14.2)	44 (9.9)	56 (21.6)	**<0.001**
Hemodyalysis	58 (8.3)	24 (5.4)	34 (13.1)	**<0.001**
Severity Scores				
APACHE-II scores	19.97 ± 8.30	17.11 ± 7.53	24.86 ± 7.21	**<0.001**
SOFA scores	5 (3–8)	4 (2–7)	8 (5–10)	**<0.001**
CCI	5 (2–9)	4 (1–8)	7 (4–10)	**<0.001**
IMV treatment	355 (50.6)	178 (40.2)	177 (68.3)	**<0.001**
Duration of IMV (days)	10 (4–24)	16 (3–39)	8 (5–15)	**<0.001**
Length of stay in ICUs (days)	8 (5–18)	7 (4–23)	9 (5–18)	0.263
Length of stay in hospital (days)	15 (8–25)	15 (10–33)	14 (7–22)	**<0.001**
90-day mortality	332 (47.3)			

Note. COPD: Chronic Obstructive Pulmonary Disease, IMV: Invasive Mechanical Ventilation, APACHE-II: Acute Physiologic and Chronic Health Evaluation-II, SOFA: Sequential Organ Failure Assessment, CCI: Charlson Comorbidity Index, ICUs: Intensive Care Units. Statistically significant *p*-values are shown in bold.

**Table 2:** Laboratory values of the patients at admission; comparison between survivors and non-survivors.

	All Patients n:702 (100%)	Survivors n:443 (63.1%)	Non-Survivors n:259 (36.9%)	*p*-Value
Hemoglobin (g/dL)	10.90 (9.40–12.60)	11.10 (9.70–13.00)	10.50 (9.00–12.10)	**<0.001**
Leukocyte (10^3^/μL)	12.51 (8.83–17.31)	11.94 (8.70–16.52)	13.42 (9.22–18.12)	**0.009**
Neutrophil (10^3^/μL)	10.69 (7.02–16.33)	9.94 (6.71–14.26)	11.91(7.92–16.73)	**<0.001**
Lymphocyte (10^3^/μL)	0.90(0.56–1.40)	0.95 (0.65–1.48)	0.79 (0.42–1.22)	**<0.001**
Platelet (10^3^/μL)	228 (166–304)	234 (180–305)	205 (139–300)	**0.001**
INR	1.15 (1.06–1.30)	1.12(1.04–1.25)	1.24 (1.10–1.47)	**<0.001**
aPTT (second)	29.55 (26.40–34.70)	28.80 (26.02–32.80)	32.05 (27.17–38.80)	**<0.001**
Glucose (mg/dL)	148 (117–194)	146 (117–187)	151 (120–208)	0.216
Creatinine (mg/dL)	0.99 (0.69–1.62)	0.87 (0.65–1.23)	1.23 (0.83–2.27)	**<0.001**
Sodium (mmol/L)	139 (136–142)	139 (136–142)	138 (135–143)	0.200
Potassium (mmol/L)	4.20 (3.70–4.80)	4.20 (3.80–4.60)	4.20 (3.60–5.00)	0.409
Chlorine (mmol/L)	103 (99–107)	103 (100–107)	102 (98–108)	**0.047**
a-c Calcium (mg/dL)	9.52 (8.94–10.28)	9.44 (8.92–10.10)	9.74 (8.96–10.44)	**0.001**
Magnesium (mg/dL)	1.93 (1.71–2.20)	1.90 (1.68–2.14)	2.01 (1.79–2.29)	**0.001**
Phosphorus (mg/dL)	3.60 (2.90–4.50)	3.50 (2.90–4.20)	3.90 (3.10–5.30)	**<0.001**
AST (U/L)	33 (21–63)	30 (20–55)	39 (23–87)	**<0.001**
ALT (U/L)	20 (12–39)	19 (12–36)	22 (13–46)	**0.034**
Total bilirubin (mg/dL)	0.57 (0.36–0.94)	0.57 (0.37–0.86)	0.57 (0.36–1.15)	0.266
Direct bilirubin (mg/dL)	0.27 (0.16–0.49)	0.25 (0.15–0.40)	0.30 (0.19–0.74)	**<0.001**
Total Protein (g/dL)	5.66 (4.98–6.39)	5.77 (5.08–6.46)	5.46 (4.81–6.24)	**0.001**
Albumin (g/dL)	3.10 (2.50–3.60)	3.20 (2.70–3.70)	2.80 (2.30–3.30)	**<0.001**
CRP (mg/L)	65.00 (16.57–158.11	50.70 (10.21–143.00)	92.01 (24.30–179.06)	**<0.001**
Procalcitonin (µg/L)	0.42 (0.13–1.80)	0.26 (0.009–0.89)	0.86 (0.27–3.9)	**<0.001**
pH	7.39 (7.31–7.45)	7.40 (7.34–7.45)	7.36 (7.27–7.44)	**<0.001**
Lactate (mmol/L)	1.71 (1.22–2.57)	1.55 (1.13–2.22)	2.03 (1.44–3.04)	**<0.001**

Note. INR: International Normalized Ratio, aPTT: activated Partial Thromboplastin Time, a-c Calcium: albumin-corrected Calcium, AST: Aspartate Amino-Transferase, ALT: Alanine Amino-Transferase, CRP: C-Reactive Protein, pH: power of Hydrogen. Statistically significant *p*-values are shown in bold.

**Table 3:** Comparison of inflammatory parameters among survivors and non-survivors.

Inflammatory Parameters	All Patients n:702 (100%)	Survivors n:443(63.1)	Non-Survivors n:259 (36.9%)	*p*-Value
SII	2573.19 (1257.06–4805.89)	2461.73 (1193.89–4295.11)	2890.33 (1392.34–6645.25)	**0.010**
NLR	11.55 (6.18–20.89)	10.42 (5.62–17.11)	15.86 (6.96–28.59)	**<0.001**
PLR	243.74 (145.94–412.28)	243.10 (146.60–397.56)	259.09 (147.05–444.23)	0.326

Note. SII: Systemic Inflammation Index, NLR: Neutrophil/Lymphocyte Ratio, PLR: Platelet/Lymphocyte Ratio. Statistically significant *p*-values are shown in bold.

**Table 4:** Multivariate logistic regression analysis for risk factors for mortality.

Risk Factors	OR (95% CI)	*p* Value
APACHE-II score	1.065 (1.028–1.102)	**<0.001**
SOFA	1.202 (1.107–1.304)	**<0.001**
CCI	1.111 (1.064–1.160)	**<0.001**
CRP (mg/L)	1.002 (1.000–1.004)	0.051
Lactate (mmol/liter)	1.156 (1.053–1.269)	**0.002**
Creatinine (mg/dL)	1.097 (0.937–1.283)	0.251
SII/1000	1.029 (1.001–1.057)	**0.042**

Note. APACHE-II: Acute Physiologic and Chronic Health Evaluation-II, SOFA: Sequential Organ Failure Assessment, CCI: Charlson Comorbidity Index, CRP: C-Reactive Protein, NLR: Neutrophil/Lymphocyte Ratio, SII: Systemic Inflammation Index, OR: Odds ratio, CI: Confidence Interval. Statistically significant *p*-values are shown in bold.

## Data Availability

Data is available upon request to the corresponding author. It is not publicly available due to confidentiality reasons.
